# Early Effector CD8 T Cells Display Plasticity in Populating the Short-Lived Effector and Memory-Precursor Pools Following Bacterial or Viral Infection

**DOI:** 10.1038/srep12264

**Published:** 2015-07-20

**Authors:** Courtney R. Plumlee, Joshua J. Obar, Sara L. Colpitts, Evan R. Jellison, W. Nicholas Haining, Leo Lefrancois, Kamal M. Khanna

**Affiliations:** 1 Dept. of Immunology, University of Connecticut Health Center, Farmington, CT; 2 Dept of Pediatrics, University of Connecticut Health Center, Farmington, CT; 3 Dept. of Immunology & Infectious Disease, Montana State University, Bozeman, MT; 4 Dept. of Pediatric Oncology, Dana-Farber Cancer Institute, Boston, MA

## Abstract

Naïve antigen-specific CD8 T cells expand in response to infection and can be phenotypically separated into distinct effector populations, which include memory precursor effector cells (MPECs) and short-lived effector cells (SLECs). In the days before the peak of the T cell response, a third population called early effector cells (EECs) predominate the antigen-specific response. However, the contribution of the EEC population to the CD8 T cell differentiation program during an antimicrobial immune response is not well understood. To test if EEC populations were pre-committed to either an MPEC or SLEC fate, we purified EECs from mice infected with *Listeria monocytogenes* (LM) or vesicular stomatitis virus (VSV), where the relative frequency of each population is known to be different at the peak of the response. Sorted EECs transferred into uninfected hosts revealed that EECs were pre-programmed to differentiate based on early signals received from the distinct infectious environments. Surprisingly, when these same EECs were transferred early into mismatched infected hosts, the transferred EECs could be diverted from their original fate. These results delineate a model of differentiation where EECs are programmed to form MPECs or SLECs, but remain susceptible to additional inflammatory stimuli that can alter their fate.

Cytotoxic CD8 T cells play an important role in controlling intracellular infections through recognition of antigenic peptides presented on MHC class I molecules. As few as 80–1,200 naive antigen-specific CD8 T cells expand in response to the combination of TCR triggering and inflammatory stimuli to form a large effector T cell population capable of controlling intracellular pathogens^1^,^2^. Following expansion, the majority of the antigen-specific CD8 T cells die, leaving behind a small memory population that is capable of surviving long-term and protecting the host from subsequent challenge^3^. Memory CD8 T cells rapidly expand and re-express effector molecules, such as granzyme B and IFN-γ, upon re-exposure to antigen for efficient control of the secondary infection^4^.

At the peak of the T cell response to an acute infection (~7–9 days post-infection) a subset of CD11a^high^, CD8 T cells upregulate or retain IL-7 receptor α (IL-7Rα or CD127) expression, and this subset of cells subsequently forms the functional memory CD8 T cell pool^5^,^6^. Expression of killer-cell lectin-like receptor G1 (KLRG1) is also used to further differentiate the total population of CD8 T cells that are present at this time^7^,^8^. Based on the expression of these two receptors, CD8 T cells can be separated into a memory precursor effector cell (MPEC) population which expresses CD127, but not KLRG1 (CD127+ KLRG1−) and a short lived effector cell (SLEC) population which expresses KLRG1, but not CD127 (CD127− KLRG1+)^9^. Our lab previously phenotyped CD8 T cells prior to the peak of the immune response (2–5 days post-infection) and showed that the majority of cells lack expression of both CD127 and KLRG1 (CD127-KLRG1-) at this time^10^. This population has the capacity to give rise to both SLECs and MPECs. These early effector cells (EECs) are so named because on par with SLECs and MPECs, they express the effector molecules granzyme B and IFN-γ early after infection^4^.

Different infections and inflammatory environments have drastic effects on the differentiation of effector CD8 T cells^10^,^11^,^12^,^13^,^14^. IL-12 and IL-2 have an important role in the differentiation of SLECs by regulating transcription factors such as T-bet and Blimp1^7^,^11^,^15^,^16^,^17^. More recently, the transcription factors ID3 and STAT3 have been shown to drive MPEC generation^18^,^19^. The metabolic status of the CD8 effector cell can also influence differentiation through the mTOR pathway^20^,^21^,^22^. Although we have previously identified cytokines and transcription factors that regulate the differentiation of antigen specific CD8 T cell differentiation into SLEC and MPEC^10^, it remains unclear how stable these differentiation events are and how early these events are predetermined. Thus, while both SLECs and MPECs arise from the EEC population, it remains unclear if commitment towards a SLEC versus MPEC fate occurs as cells transition through the EEC phase.

To address these questions, we transferred purified EECs into uninfected recipients and showed that the signals received at priming continue to drive differentiation towards SLECs or MPECs. To test plasticity, we transferred EECs into mismatched infections and demonstrated that, at the population level, they retain the ability to form either SLECs of MPECs. These results suggest that EECs are imprinted during priming but still retain plasticity to respond to specific external inflammatory cues that are induced by the type of invading pathogen.

## Results

### EEC frequency varies between infections

**Figure 1:**
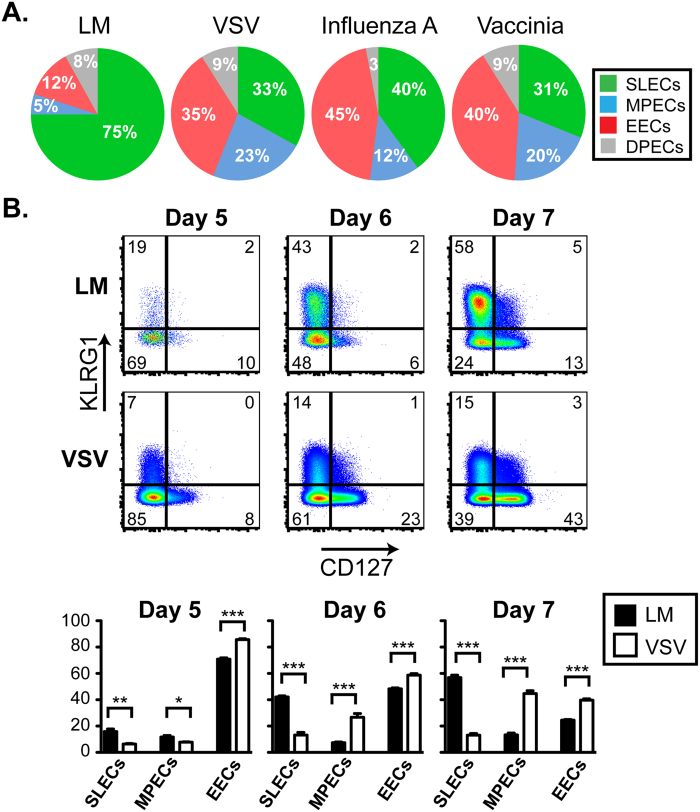
EEC emerge following multiple infection early after infection. **A**. OVA-specific CD8 T cells were identified as: CD8+, OVA MHC class I tetramer+, CD11a^hi^ within the splenocytes at the peak of the T cell response to LM-OVA (day 8), VSV-OVA (day 7), Influenza A-OVA (day 10), and Vaccinia-OVA (day 7) and analyzed for KLRG1 and CD127 expression. In the pie charts, KLRG1+ CD127− cells are classified as SLECs, KLRG1− CD127+ as MPECs, KLRG1− CD127− as EECs and KLRG1+ CD127+ as DPECs for each infection. **B**. 1,000 CD45.1+ transgenic OT-I T cells were transferred to uninfected CD45.2+ recipients one day prior to infection with LM-OVA or VSV-OVA. At days 5, 6 and 7 post-infection, CD45.1+ OT-I T cells were analyzed in the spleen for KLRG1 and CD127. Percentages of SLECs, MPECs, and EECs from each condition are graphed from 3–4 mice. This data is representative of at least 2 experiments.

While MPECs and SLECs have been studied extensively, much less is known about the role and presence of EECs following infection. We performed studies to track the effector CD8 T cell populations after infection with *Listeria monocytogenes* (LM), vesicular stomatitis virus (VSV), influenza A virus (IAV), or vaccinia virus (VV). In these studies we infected mice with pathogens expressing the model antigen ovalbumin (OVA) in order to measure the response of OVA-specific cells within the endogenous pool of CD8 T cells using MHC class I tetramers. Using the lack of CD127 and KLRG1 expression to identify EECs, our results indicated that the frequency of EECs present at the peak of the immune response was different in the spleens of mice infected with LM versus VSV (Fig. 1A). The response to LM was dominated by SLEC generation with few EECs present at the peak. Alternatively, EECs were present at a higher frequency than either SLECs or MPECs alone following VSV infection. Similar to VSV, infection with IAV or VV, which both express OVA, induced a robust population of antigen-specific EECs at the peak of the T cell response (Fig. 1A).

The endogenous response can also be mimicked using the transfer of low numbers of OVA-specific OT-I cells from transgenic mice to congenic wild-type recipients^23^. When mice were infected with LM-OVA or VSV-OVA one day following cell transfer, we found a considerable difference in the percentage of EECs between the two infections (Fig. 1B). At 7 days post-infection, the pattern of OT-I differentiation was similar to that of the endogenous response with LM driving more SLECs and VSV inducing more EECs and MPECs. However, transgenic OT-I cells exhibited a higher propensity for forming MPECs than endogenous OVA-specific CD8 T cell, which was observed for both LM and VSV infections. Importantly, when we examined earlier time points following infection, we found that the majority of OT-I cells progressed through an EEC phase (day 5 post-infection) (Fig. 1B), which was also observed for the endogenous population of OVA-specific cells. Thus, our results suggest that the EEC population represents a transitory state of differentiation from which downstream subsets can emerge. In addition, the nature of the inflammatory environment during infection can influence the long-term survival or retention of this population.

To further phenotype the EEC population, we analyzed the expression of additional receptors and transcription factors from both LM and VSV EECs 5 days after infection (Supplementary Figure S1). The expression of cell surface markers CD44, CD69, Ly6C and CD62L was very similar in both populations of EECs. Only CD25 showed substantial differential expression between the two populations. This was expected based on the results from a previous paper which demonstrated that CD25 peaks earlier during VSV infection (Day 3–4) and late IL-2 signaling is most critical for the SLEC subset^24^. Ki-67, a cellular marker for proliferation was identical between LM and VSV EECs as were transcription factors T-bet, Bcl-6, and Eomes, all of which have been shown to play a role in CD8 T cell differentiation^1^. Overall, the EEC population from both LM and VSV infected animals appears to be a homogeneous population with remarkably few differences between the two infections.

### EECs continue to follow differentiation cues in the absence of inflammation.

**Figure 2:**
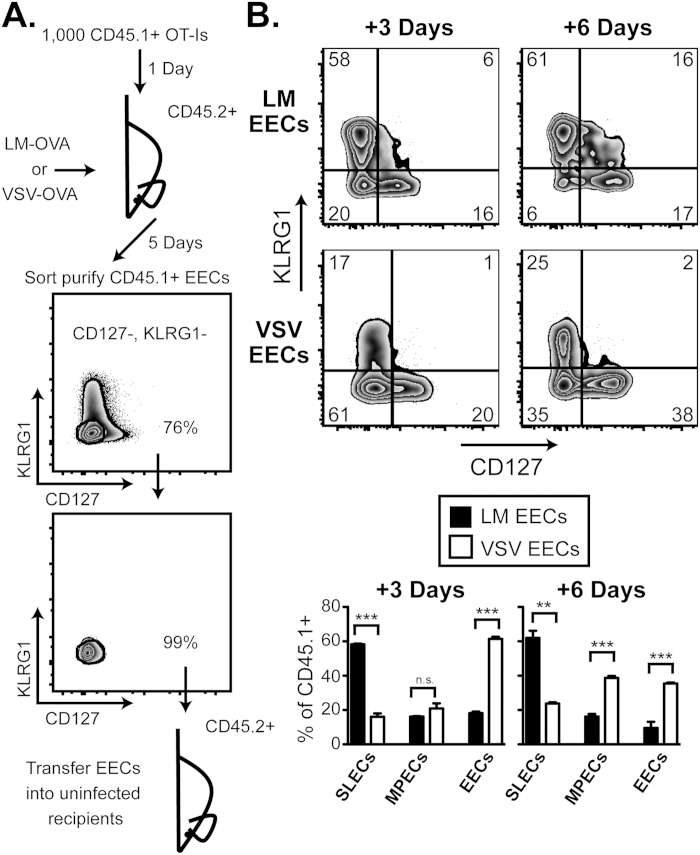
EECs differentiate based on previously received inflammatory signals in uninfected recipients. **A**. Schematic of EEC sorting strategy. 1,000 CD45.1+ OT-I cells were transferred to uninfected CD45.2+ recipients one day prior to infection with LM-OVA or VSV-OVA. 5 days following infection, CD8+ CD45.1+ KLRG1− CD127− EECs were sorted to ~99% purity. **B**. EECs were purified, as described in A, from both LM-OVA- and VSV-OVA-infected hosts and transferred to uninfected recipients. CD8+ CD45.1+ transferred EECs were then analyzed for KLRG1 and CD127 expression from splenocytes 3 or 6 days post-transfer. Percentages of SLECs, MPECs, and EECs from each condition are graphed from 3–4 mice. This data is representative of at least 2 experiments.

In order to determine the differentiation potential and commitment of EECs, purified EECs were transferred to recipient mice and their fate was examined under different conditions. To facilitate the sorting of EECs without using MHC class I tetramers, which may engage the TCR thus activating the cells, low numbers (1,000) of CD45.1+ OT-I cells were transferred into CD45.2+ C57Bl/6 mice one day prior to infection with LM-OVA or VSV-OVA. As shown in Fig. 2A, 5 days following infection, CD8 CD45.1+ KLRG1− CD127− EECs were sorted to 99% purity and transferred again into naive CD45.2+ recipients.

We first transferred EECs into uninfected recipients to test the differentiation capacity of this population in the absence of additional antigen or extrinsic inflammatory cues. We found that LM EECs transferred into uninfected mice and left for an additional 3 or 6 days differentiated into SLECs and MPECs (Fig. 2B), which suggested that EECs are a source of downstream MPEC and SLEC populations. Moreover, consistent with the response of endogenous CD8 T cells, there was an approximately 3-fold greater SLEC population than MPEC population at both time points reflecting LM’s propensity for inducing SLEC generation. EECs taken from VSV-infected mice showed a significantly different phenotype after 3 or 6 days in uninfected recipients with more MPECs generated than SLECs, as well as retention of EECs (Fig. 2B). These results suggest that EECs are pre-programmed to form MPECs or SLECs as early as 5 days after infection and continue to develop along this differentiation pathway in the absence of continued TCR engagement and inflammation.

Additionally, we wanted to test if manipulating the inflammatory cytokine milieu during a VSV infection would skew the EECs to preferentially differentiate into SLECs. To this end, we infected mice with VSV and 90 minutes later, treated the animals with 50 ug ODN 1826, a type B CpG-containing oligonucleotide, that induces large amounts of IL-12^10^. Previous studies have shown that mice infected with VSV and treated with ODN 1826 have increased formation of SLECs within the antigen specific CD8 T cell population^10^. Thus, to determine if ODN 1826 could influence EEC differentiation, we transferred Day 5 EECs from VSV infected mice, with or without ODN 1826 treatment, into uninfected recipients. 3 days following transfer, EECs that came from mice treated with ODN 1826 differentiated into significantly more SLECs in the uninfected recipients (Supplementary Figure S2). These results demonstrated that additional production of inflammatory cytokines, during VSV infection, was sufficient to imprint a greater percentage of EECs to differentiate into SLECs, much like EECs taken from LM infected animals produce more SLECs.

### VSV EECs exhibit an enrichment of MPEC genes by microarray analysis.

**Figure 3:**
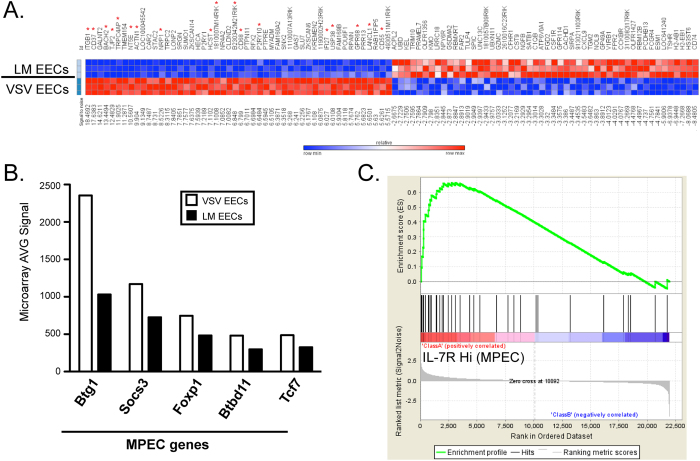
Whole genome microarray studies reveal a subset of genes differentially expressed in EECs generated after LM versus VSV infection. **A**. mRNA Expression levels were measured by microarray analysis with Illumina BeadChips of sorted EECs, as in Fig. 2A, 5 days following LM-OVA or VSV-OVA infection. The top 50 and bottom 50 differentially regulated genes, ranked by signal-to-noise, are displayed. Each row represents an individual sample. **B**. The AVG signal from the microarray of LM or VSV EECs for selected MPEC genes is graphed. **C**. Enrichment profile generated from GSEA using the top 50 genes enriched in VSV EECs compared to a gene set generated with IL-7R^Hi^ (MPEC) and IL-7R^Lo^ (SLEC) samples. Genes identified as being enriched in both VSV EECs and IL-7R^Hi^ cells (MPECs) are marked with a red asterisk in part A.

Our results suggested that EECs were committed to a downstream fate based on signals received in different infectious environments. Therefore, we examined genetic differences between VSV and LM EECs to identify genes enriched within VSV EECs that may be important for MPEC generation and genes enriched within the LM EECs that may be important for SLEC differentiation. RNA was isolated from purified EECs sorted from either VSV- or LM- infected mice, and used for whole-genome analysis with Illumina BeadChips. We found 53 genes that were 2-fold higher in VSV EECs compared to LM EECs and 63 genes at least 2-fold higher in LM EECs compared to VSV EECs. Figure 3A shows the top 50 upregulated or downregulated genes, ranked by the signal-to-noise metric. We first looked at genes from the literature known to be important for MPEC or SLEC development^1^,^25^. Figure 3B shows an example of 5 MPEC genes that were enriched in VSV EECs, which hints at a correlation between VSV EECs and MPECs.

To further identify unknown MPEC- or SLEC-specific genes in VSV or LM EECs, we used gene set enrichment analysis (GSEA) to compare our results with a previously published microarray which sorted IL-7R^lo^ (SLEC) and IL-7R^hi^ (MPEC) transgenic P14 CD8 T cells 6-7 days following lymphocytic choriomeningitis virus (LCMV) infection^7^,^26^. Using this IL-7R^lo^ versus IL-7R^hi^ array to make a ranked list of genes, we then made VSV EEC- and LM EEC-specific gene sets of the top 25, 50, 100, or 200 genes based on signal-to-noise. Using GSEA to compare the gene sets from EECs to the ranked list of IL-7R^lo^ (SLECs) versus IL-7R^hi^ (MPECs), we found a statistically significant correlation with the 25, 50, 100, and 200 top genes from VSV EEC to the IL-7R^hi^ (MPEC) phenotype. An example of the enrichment plot for the top 50 genes from VSV EECs is shown in Fig. 3C, and the genes found in both arrays are highlighted with red asterisks in Fig. 3A. Additionally, the 50 most upregulated or downregulated genes between VSV EECs and LM EECs, ranked by signal to noise metric, is shown in Supplementary Table S1. This result indicates that IL-7R^hi^ or MPEC-specific genes are enriched within VSV EECs before they have differentiated and are predictive of the resulting downstream phenotype. Thus, MPEC genes expressed at the EEC stage may drive differentiation towards the memory precursor fate. Interestingly, the same trend was not observed for SLEC genes in LM-EECs for unknown reasons.

### EECs exhibit plasticity when exposed to disparate inflammatory conditions.

**Figure 4:**
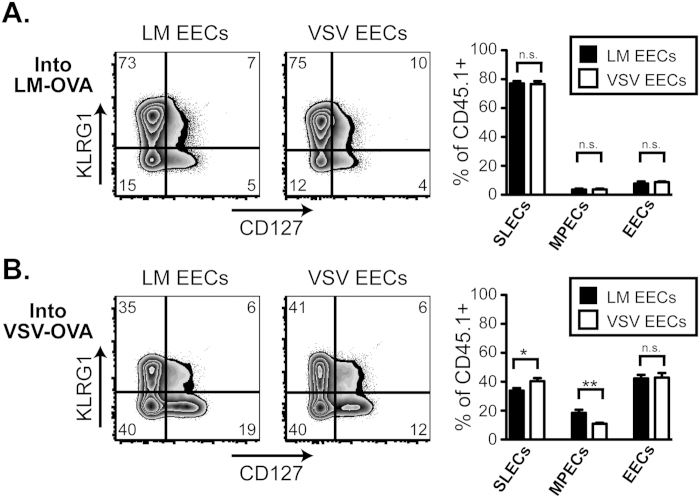
EECs transferred into infected mice show plasticity by modulating their differentiation according to the infection-induced inflammatory environment of the recipient mouse. **A**. EECs were purified, as described in Fig. 2A, following LM-OVA or VSV-OVA infection and transferred into LM-OVA infected recipients that had been infected for 2 days. 6 days following the EEC transfer, CD8+ CD45.1+ transferred EECs in the spleen were analyzed for KLRG1 and CD127. Percentages of SLECs, MPECs, and EECs are graphed for both LM-OVA EECs and VSV-OVA EECs from 3–4 mice. This data is representative of at least 2 experiments. **B**. Same as A, except recipient mice were infected with VSV-OVA 2 days prior to EEC transfer.

In the absence of inflammation, EECs proved committed to a fate initially programmed by the infectious setting. To test if the commitment of EECs towards an MPEC or SLEC fate was maintained in the presence of additional exogenous stimuli, EECs were again purified 5 days after LM-OVA or VSV-OVA infection (as in Fig. 2A), but sorted cells were transferred into recipients that had been infected with LM-OVA or VSV-OVA 2 days before. Under these conditions both antigen and inflammation are high in the recipient mice. The transferred cells were then examined 6 days after transfer in the infected recipients (i.e. day 8 after initial infection). Unlike our earlier results, both LM EECs and VSV EECs formed robust populations of SLECs when transferred into LM-infected recipients (Fig. 4A). The opposite was found when LM EECs or VSV EECs were transferred into VSV-infected recipients (Fig. 4B) where both formed significantly more EECs and MPECs (compared to the LM recipients), with LM EECs forming slightly more MPECs. In both conditions, the source of the EECs had little to no effect on the downstream phenotype of the transferred OT-I CD8 T cells. Conversely the environmental setting driven by the type of infection in the recipient animals was critical for regulating cellular differentiation. These results suggest that although EECs may be pre-programmed to form SLECs or MPECs when transferred into uninfected recipients, their capacity to form either subset remains intact when provided the necessary environmental signals to re-direct differentiation.

### Antigen is required for optimal differentiation and expansion of EECs

**Figure 5:**
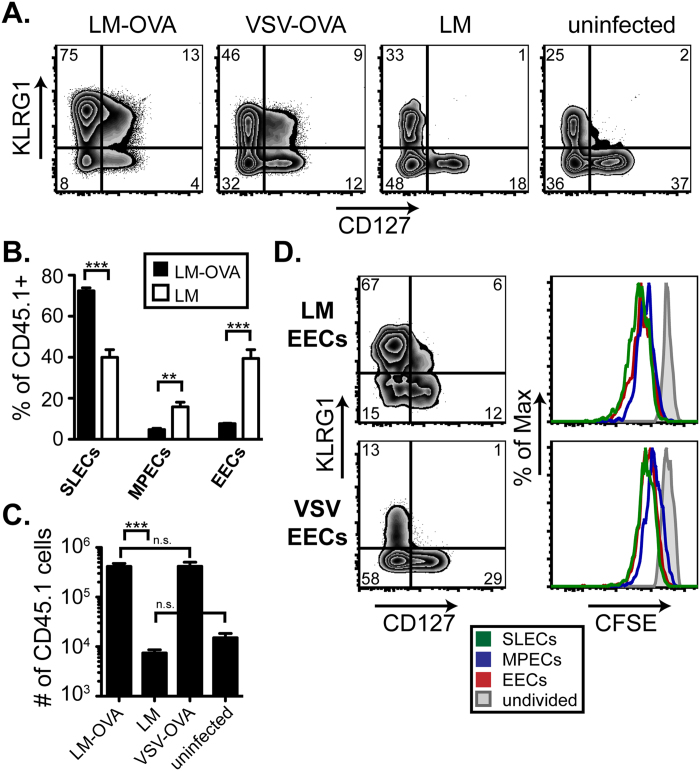
Optimal SLEC generation and proliferation requires continued antigen expression. **A**. EECs were purified, as described in Fig. 2A, following VSV-OVA infection and transferred to recipient mice infected with LM-OVA, VSV-OVA, LM or left uninfected. 6 days following transfer, the CD8+, CD45.1+ EECs in the spleen were analyzed for KLRG1 and CD127 expression. Each condition was repeated at least 2 times. **B**. Percentages of SLECs, MPECs, and EECs are graphed for both LM-OVA EECs and LM EECs from 3–4 mice. **C**. Total numbers of CD8+ CD45.1+ transferred EECs per spleen, from the experiment in part A are shown graphically. **D**. EECs from both VSV-OVA- and LM-OVA-infected mice were sort purified as in Fig. 2A. Following purification, CD45.1+ EECs were mixed with CD45.2+ splenocytes from a naïve mouse at a 1:10 ratio prior to CFSE labeling. Total CFSE labeled cells were then transferred to uninfected recipients and CD8 CD45.1+ EECs were analyzed for KLRG1 and CD127 expression and CFSE dilution 3 days following transfer. The CFSE labeled CD45.2+ splenocytes were used as the undivided control.

Since we found that EECs retained the capacity to change their fate in certain environments, we further dissected the role of antigen versus inflammation in SLEC or MPEC differentiation by utilizing a strain of LM that does not express OVA. Again, VSV EECs were transferred into infected recipients (2 days post-infection), but recipient mice were infected with LM-OVA, VSV-OVA or LM without OVA versus uninfected controls. As discussed above, after 6 days VSV EECs transferred into LM-OVA-infected recipients predominantly formed SLECs, while VSV EECs transferred into VSV-OVA-infected recipients generated more EECs and MPECs (Fig. 5A). When the VSV EECs were transferred into LM-infected recipients (lacking OVA expression), the phenotype dramatically changed from that of the LM-OVA-infected recipients. Only 33% of the EEC-derived population exhibited a SLEC phenotype in LM-infected recipients compared to 75% in LM-OVA recipients (Fig. 5B). Additionally, the overall expansion of the EECs transferred into LM-infected recipients was significantly reduced compared to LM-OVA-infected recipients (Fig. 5C). Interestingly, while reduced, the expansion of EECs in the presence of inflammation but absence of antigen was more similar to the expansion observed when EECs were transferred into uninfected recipients lacking both inflammation and antigen (Fig. 5C). Thus, despite the presence of similar inflammatory factors in the two groups of LM-infected recipients, the antigen was clearly required for optimal expansion and differentiation into SLECs. Moreover, the EECs transferred into LM-infected recipients showed a reduction in MPEC generation when compared to the uninfected recipients (18% vs 37%) suggesting a role for inflammation in limiting entry into the MPEC compartment (Fig. 5A).

After observing a difference in the overall expansion of EECs in different settings, we wanted to further investigate potential proliferative differences between the individual effector subsets during differentiation. Therefore, we labeled EECs with CFSE prior to transfer into uninfected recipients. As discussed in Fig. 2B, LM EECs continued to differentiate into SLECs in uninfected recipients, while VSV EECs formed more MPECs or EECs. When the extent of CFSE dilution of each effector subset was analyzed, no major differences in proliferation were observed after 3 days (Fig. 5D). It appeared that all of the subsets had divided once or twice when compared to the undivided control. Therefore early proliferation differences did not account for the different frequencies of effector subsets derived from LM EECs versus VSV EECs.

### With time, EECs lose their ability to differentiate without re-stimulation

**Figure 6:**
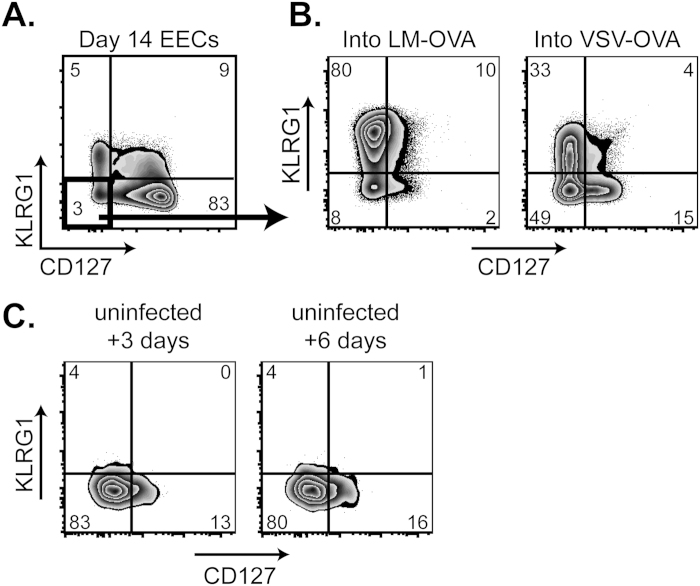
With time, EECs lose their ability to generate MPEC and SLEC in the absence of exogenous inflammatory stimuli. **A**. 14 days following VSV-OVA infection, EECs were purified as in Fig. 2A and transferred into day 2 LM-OVA- or VSV-OVA-infected recipients. 6 days following EEC transfer, CD8 CD45.1+ EECs were analyzed for KLRG1 and CD127 expression. Each condition was repeated at least 2 times. **B**. Day 14 purified EECs from VSV-OVA infection were transferred into uninfected recipients, and CD8 CD45.1+ EECs in the spleen were analyzed for KLRG1 and CD127 expression 3 or 6 days following transfer. Each condition was repeated at least 2 times.

In all of the experiments described above, EECs were sorted 5 days following infection when a large proportion of the antigen-specific cells have this phenotype. Although the population of EECs decreases considerably with time (Fig. 1A and Fig. 6A), we wanted to examine if EECs maintained later in the ongoing immune response still retained their ability to differentiate into SLECs or MPECs. We generated EECs in VSV-OVA infected animals, as in previous experiments, but waited until 14 days after infection to purify and transfer the sorted EECs into new recipients that were infected with either LM-OVA or VSV-OVA. The EECs retained the capacity to robustly differentiate into all of the effector subsets, with LM infection driving plentiful SLEC differentiation and VSV infection driving more MPECs and EECs (Fig. 6B). Interestingly, day 14 VSV EECs, when transferred into uninfected recipients, remained almost entirely EECs 3 or 6 days after transfer (Fig. 6C). These results suggested that as late as 14 days after infection, a small percentage of antigen-specific CD8 T cells that retained the EEC phenotype were still present and failed to exhibit pre-programmed differentiation into SLECs and MPECs.

## Discussion

Previous publications from our lab have shown that VSV versus LM infection results in increased EECs at the peak of the T cell response, and that at early time-points for both infections, virtually all the antigen-specific CD8 T cells have an EEC phenotype^10^,^24^. Because EECs are the source of both MPECs and SLECs, we wanted to understand the precise contribution of the EEC population to the CD8 T cell differentiation program during the progression of an antimicrobial immune response. Thus, in the current study, we determined if commitment to a memory lineage is designated at the EEC stage and if EECs represent a heterogeneous population of cells with respect to their differentiation potential. We began by exploring precisely at what point an EEC becomes committed to a particular lineage or if the population remains malleable and retains the capacity to differentiate into other populations. We discovered that EECs transferred into naïve mice (which lack antigen and an inflammatory milieu) were capable of differentiating into a heterogeneous population of effector CD8 T cells. Importantly, the EECs purified from LM- compared to VSV-infected animals showed clear diversity in their differentiation pattern, with EECs from LM-infected mice forming more SLECs and the EECs from VSV-infected mice forming more MPECs. This implied that the transferred EECs were already imprinted towards a particular fate during priming in the host, likely by pro-inflammatory cytokines and antigen and could continue down the same path after transfer into naïve recipients in the absence of additional inflammatory or antigenic signals.

To better understand this imprinting, we utilized ODN 1826 CpG treatment to supplement VSV infection with additional IL-12 and pro-inflammatory cytokines. When ODN 1826 was administered after VSV infection, the EECs sorted from these mice differentiated into significantly more SLECs than EECs sorted from VSV-infected mice without ODN 1826 treatment. For this experiment, it is likely that the priming environment would be similar, except for the additional cytokines from ODN 1826 treatment, however, the increased cytokine production may indirectly affect viral replication. This result again demonstrated that the inflammatory milieu, present during EEC development, was responsible for driving differentiation, even after transfer into uninfected recipients, probably by IL-12 dependent mechanisms^10^.

A majority of the previous microarray studies have either compared gene expression profiles of naïve, effector and memory CD8 T cells following infection^25^,^27^,^28^,^29^,^30^ or have analyzed genetic differences between effector subsets, but these studies were done after differentiation into MPECs or SLECs had already occurred^7^,^8^. Thus, in this study, we investigated genetic factors in EECs generated following VSV versus LM infection that are responsible for driving differentiation towards an MPEC or SLEC fate. Although both types of EECs (i.e. after VSV or LM infection) represented early effector CD8 T cells that exhibited an identical phenotype (i.e. CD127-, KLRG1-), there were still over 100 genes that were more than 2-fold differentially expressed between VSV and LM EECs. When we used GSEA to compare gene expression profiles of VSV vs LM EECs to those of MPECs or SLECs, we were able to observe a clear enrichment of MPEC (IL-7R^hi^) related genes within the VSV EECs. These data suggest that genes expressed in MPECs may be turned on during the EEC stage to drive differentiation towards the memory precursor fate. Future studies exploring the precise role of these individual genes will be important to develop more effective vaccines that favor the generation of MPECs.

Two recent studies that examined CD8 effector T cell differentiation at the single cell level following infection^31^,^32^ found extreme heterogeneity between different CD8 T cell clones in their ability to form MPECs or SLECs. Work from our lab further demonstrated that the infectious environment in which the clones develop heavily influenced their fate^33^. However, whether an early effector CD8 T cell retains plasticity in the type of effector cell it will become or if this fate is fixed remains unknown. Thus, to test the true plasticity of EECs, we transferred phenotypically identical EECs from LM- or VSV-infected mice into infection matched or mis-matched recipients. If EECs were committed to their fate, the infection type of the recipient mice would be irrelevant in determining the outcome of the cells. Instead, we discovered that the differentiation pattern of the transferred EECs was heavily influenced by the infectious milieu of the recipient animal. For example, both EECs from LM and VSV infections, when transferred into LM-infected recipients, heavily skewed towards the formation of SLECs. This result is in contrast to the asymmetric division model where asymmetric division leads to the development of distinct effector and memory CD8 T cell lineages^34^. Instead, we propose that plasticity exists in EECs, which allows ongoing molding of the differentiation program of the CD8 T cell response well downstream of initial division. While this scenario does not preclude the asymmetric model of memory CD8 T cell development, we believe that our studies will add to the current paradigm explaining the early events surrounding effector and memory CD8 T cell development.

To determine the influence of antigen and/or inflammation, we transferred OVA-specific EECs into recipients who had been infected 2 days previously with either LM-OVA or LM lacking OVA expression. Following transfer, robust SLEC generation was not observed when VSV-generated EECs were put into LM-infected recipients, and the phenotype of the transferred EECs more closely resembled that of EECs transferred into naïve recipients. In addition, the expansion of EECs transferred into LM recipients was several logs lower than EECs transferred into LM-OVA recipient and, again, closely resembled EECs transferred into naïve recipients. These data demonstrate that continued antigen exposure, likely in concert with inflammatory cytokines, is necessary for efficient differentiation and expansion of cells after infection, which had been previously shown for SLEC generation in a peptide-pulsed dendritic cell vaccination model^11^.

The proportion of SLECs or MPECs present at any given time could be regulated by the differentiation program or also by expansion and contraction. However, our results clearly demonstrate that the proliferation potential of SLEC and MPEC populations early after infection was identical, which suggests that LM infection drives more EECs towards a SLEC phenotype, while VSV infection propels EECs towards an MPEC phenotype. Thus, during the early stages of an antimicrobial response, genetic changes and not variable proliferation potential dictates fate decisions of early effector CD8 T cells.

In addition to day 5 EECs, we also investigated the properties of day 14 EECs. While substantially fewer EECs remain at 14 days following a VSV infection, we were able to sort purify and transfer them into LM- or VSV-infected recipients. Similar to day 5 EECs, the day 14 EECs proliferated robustly and differentiated based on the infection type of the recipient. Conversely, when the same day 14 EECs were transferred into uninfected recipients, these cells retained their EEC phenotype without differentiating any further. In this respect, the day 14 EECs behaved more like memory cells, which remain undifferentiated at steady state, but can quickly divide and differentiate when challenged.

In conclusion, in the current study, we discovered that EECs serve as master precursor effector T cells that possess a remarkable ability to differentiate into MPECs and SLECs. The nature of the differentiation pattern largely depends on antigen availability and inflammatory milieu that is induced by the type of pathogen. Thus, our study has important implications for not only understanding the basic biology of memory development but also for exploration to aid in the design of optimized pathogen- or tumor-specific CD8 T cell vaccines.

## Methods

### Mice

Female C57Bl/6 mice (CD45.2) were purchased from the National Cancer Institute (Frederick, MD) and used between 8–12 weeks of age. TCR transgenic CD45.1 OT-I Rag^-/-^ were bred and maintained in-house. The University of Connecticut Health Center Animal Care and Use Committee approved all experiments.

### Pathogens

Vesicular stomatitis virus strain expressing OVA (VSV-OVA, Indiana)^35^ and *Listeria monocytogenes* strain expressing OVA (LM-OVA)^36^ have been previously described. Infections were performed i.v. with 10^5^ pfu VSV-OVA or 10^3^ cfu LM-OVA per mouse. Intranasal infections were performed with 1 × 10^3^ pfu Influenza A virus (WSN-OVA)^37^. Vaccinia-Ova (rVV-OVA) has been previously described^38^ and was administered via skin scarification at 10^5^ pfu. Some VSV infected mice were treated with 50 ug ODN 1826 CpG (InvivoGen, San Diego, CA) i.p. in PBS, 90 minutes after infection.

### Flow Cytometry

OVA-specific T cells were stained using the H-2K^b^ tetramer containing the OVA peptide SIINFEKL generated in our lab as previously described^39^,^40^. Tetramer staining was performed for 1 hour at room-temperature. All other antibodies specific for the indicated surface molecules were stained for 20 min at 4°. Fluorescence intensities were measured on an LSR-II (BD Biosceinces, San Jose, CA) and data was analyzed using FlowJo software (Tree Star, Ashland, OR).

### Statistics

Statistical significance was determined with an unpaired, 2-tailied T-test using Prism (Graphpad, LaJolla, CA). One star indicated less than 0.05, two stars indicates less than 0.01, and three stars indicates less than 0.001.

### EEC purification and transfers

1,000 CD45.1+ OT-I cells were transferred i.v. to uninfected CD45.2+ recipients one day prior to infection with VSV-OVA or LM-OVA i.v. 5 days following infection, spleens from recipient mice were collagenase digested and stained with CD45.1 biotin, followed by anti-biotin microbeads (Miltenyi Biotec, Auburn, CA). CD45.1+ cells were enriched using an AutoMACS cell separator (Miltenyi Biotec, Auburn, CA). Following enrichment, cells were stained with CD4, MHCII, CD8, CD45.1, KLRG1, and CD127 for 20 min at 4°. CD4− MHCII− CD8+ CD45.1+ KLRG1− and CD127− EECs were sorted using a BD ARIA-II (BD Biosciences, San Jose, CA). Cell purity post-sorting was consistently 95–100%. Following sort purification, 1-2 × 10^5^ EECs were transferred i.v. to new recipient mice.

### Microarray and analysis

EECs were sort purified 5 days following either LM-OVA or VSV-OVA infection and in addition to sorting for CD45.1+ CD8+ CD127− KLRG1− cells, MHCII, CD4, and Propidium Iodide (PI) were included as a “dump” gate to exclude contaminating or dead cells. Each condition had 3 biological replicates, and in addition each biological replicate was a pooled sample from 5–10 mice. RNA was isolated using RNeasy columns (Qiagen, Valencia, CA) according to the manufacturer’s instructions. RNA was labeled and hybridized to Illumina Whole Genome Beadchips (MouseRef-8 v2, Illumina, San Diego, CA) with the help of Anu Kaur and the UConn Genomics Core. Background subtraction was performed with the Illumina Expression File Creator module within GenePattern (Broad Institute) and marker analysis was performed with GENE-E (Broad Institute). To test for enrichment of VSV or LM EEC genes within MPEC or SLEC populations, GSEA was performed as previously described^26^. We compared the top 25. 50, 100 and 200 differentially expressed genes in LM- vs. VSV- EECs to IL-7R lo and IL-7R hi gene sets from a previously published paper^7^. Microarray datasets generated in this study will be made publicly available through the National Center for Biotechnology Information Gene Expression Omnibus databases once the paper is published.

### CFSE labeling

After sorting CD45.1+ EECs, they were mixed 1:10 with CD45.2+ naïve splenocytes at a concentration of 50 × 10^6^ cells/ml in PBS. Cells were labeled using the Vybrant CFDA SE Cell Tracer Kit (Life Technologies, Grand Island, NY) at a final concentration of 5 uM. Cells were quenched with FBS, then washed 2 additional times with media. Cells were transferred i.v. to recipients and labeled CD45.2+ splenocytes were used as undivided controls.

### Ethics Statement

The University of Connecticut Health Center Animal Care and Use Committee reviewed and approved all experimental procedures. The University of Connecticut Health Center Animal Care and Use Committee adheres strictly to the guidelines for the Care and Use of Laboratory Animals of the National Institutes of Health. The committee approved the IACUC protocol number 100713-1216 after a thorough review.

## Additional Information

**How to cite this article**: Plumlee, C. R. *et al.* Early Effector CD8 T Cells Display Plasticity in Populating the Short-Lived Effector and Memory-Precursor Pools Following Bacterial or Viral Infection. *Sci. Rep.* **5**, 12264; doi: 10.1038/srep12264 (2015).

## Supplementary Material

Supplementary Information
