# Supplementary material for: Early Effector CD8 T Cells Display Plasticity in Populating the Short-Lived Effector and Memory-Precursor Pools Following Bacterial or Viral Infection
Source: Sci Rep. 2015 Jul 20;5:12264. doi: 10.1038/srep12264 (PMC4507483; doi:10.1038/srep12264)
Supplement: Supplementary Information [file srep12264-s1.pdf]

Early Effector CD8 T Cells Display Plasticity in Populating the Short-Lived Effector and Memory-Precursor Pools Following Bacterial or Viral Infection

Courtney R. Plumlee[1#], Joshua J. Obar[3], Sara L. Colpitts[1], Evan R. Jellison[1], W. Nicholas Haining[4], Leo Lefrancois[1] and Kamal M. Khanna[1,2*]

[1] Dept. of Immunology, Dept of Pediatrics, University of Connecticut Health Center, Farmington, CT

[2]Dept of Pediatrics, University of Connecticut Health Center, Farmington, CT

[3] Dept. of Immunology & Infectious Disease, Montana State University, Bozeman, MT

[4] Dept. of Pediatric Oncology, Dana-Farber Cancer Institute, Boston, MA

[#]Current Address: Seattle Biomedical Research Institute, Seattle, WA

[*] Corresponding author

kkhanna@uchc.edu (KMK)

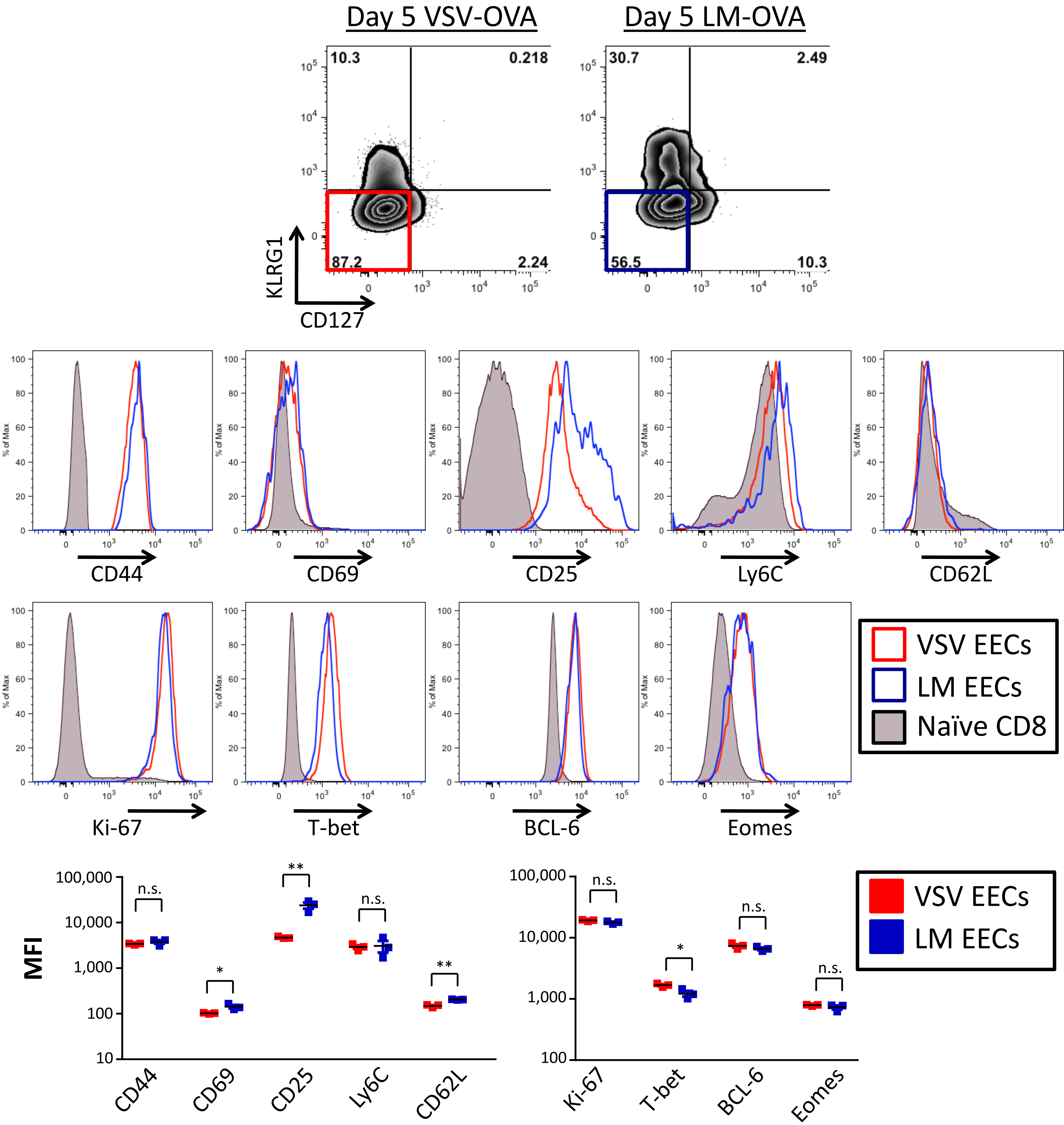

Supplemental Figure 1. Phenotyping Day 5 EECs.
1,000 CD45.1+ OT-I cells were transferred into uninfected CD45.2+ recipients one day prior to infection with VSV-OVA or LM-OVA. 5 days following infection, CD8+ CD45.1+ transferred OT-Is were analyzed for CD127 and KLRG1 expression. The CD127-, KLRG1- EECs were then further analyzed for CD44, CD69, CD25, Ly6C, CD62L, Ki-67, Tbet, Bcl-6, and Eomes expression. Histograms are show from a representative mouse of 3 with VSV EECs, LM EECs, and CD44-low naïve CD8 T cells overlaid. The Mean Fluorescence Intensity (MFI) for each marker was graphed. This experiment was repeated at least twice.

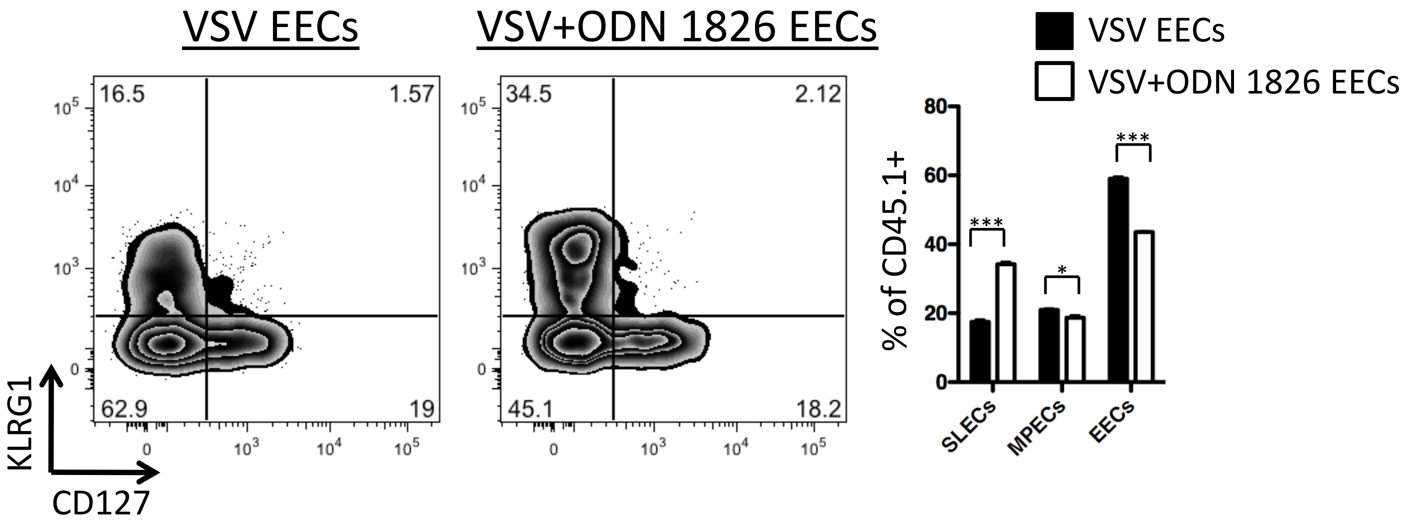

Supplemental Figure 2. ODN 1826 CpG treatment combined with VSV infection drives more SLEC differentiation.
EECs were purified, as described in Figure 2A, following VSV-OVA infection, or VSV-OVA infection with 50ug ODN 1826 CpG given i.p 90 minutes following infection and transferred into uninfected recipients. 3 days following the EEC transfer, CD8+ CD45.1+ transferred EECs in the spleen were analyzed for KLRG1 and CD127 expression. Percentages of SLECs, MPECs, and EECs are graphed for both VSV EECs and VSV+ODN 1826 CpG EECs from 3-4 mice.
